# The cost of service quality improvements: tracking the flow of funds in social franchise networks in Myanmar

**DOI:** 10.1186/1478-7547-11-14

**Published:** 2013-07-04

**Authors:** David Bishai, Amnesty LeFevre, Marc Theuss, Matt Boxshall, John D Hetherington, Min Zaw, Dominic Montagu

**Affiliations:** 1 Department of Population Family and Reproductive Health, Johns Hopkins Bloomberg School of Public Health, 615 N. Wolfe St., Baltimore, MD 21205, USA; 2 Department of International Health, Johns Hopkins Bloomberg School of Public Health, 615 N. Wolfe St., Baltimore, MD 21205, USA; 3 PSI-Myanmar, 16 West Shwe Gone Dine 4th. Street, Bahan Township, Yangon, Myanmar; 4 Department of Epidemiology, University of California San Francisco, San Francisco, California, USA

**Keywords:** Costing, Social franchise, Myanmar, Burma

## Abstract

**Introduction:**

This paper examines the cost of quality improvements in Population Services International (PSI) Myanmar’s social franchise operations from 2007 to 2009.

**Methods:**

The social franchise commodities studied were products for reproductive health, malaria, STIs, pneumonia, and diarrhea. This project applied ingredients based costing for labor, supplies, transport, and overhead. Data were gathered seven during key informant interviews with staff in the central Yangon office, examination of 3 years of payroll data, examination of a time motion study conducted by PSI, and spreadsheets recording the costs of acquiring and transporting supplies.

**Results:**

In 2009 PSI Myanmar’s social franchise devoted $2.02 million towards a 94% reduction in commodity prices offered to its network of over 1700 primary care providers. These providers retained 1/3 of the subsidy as revenue and passed along the other 2/3 to their patients in the course of offering subsidized care for 1.5 million health episodes. In addition, PSI Myanmar devoted $2.09 million to support a team of franchise officers who conducted quality assurance for the private providers overseeing service quality and to distributing medical commodities.

**Conclusion:**

In Myanmar, the social franchise operated by PSI spends roughly $1.00 in quality management and retailing for every $1.00 spent subsidizing medical commodities. Some services are free, but patients also pay fees for other lines of service. Overall patients contribute 1/6 as much as PSI does. Unlike other NGO’s, health services in social franchises like PSI are not all free to the patients, nor are the discounts uniformly applied. Discounts and subsidies evolve in response to public health concerns, market demand, providers’ cost structures as well as strategic objectives in maintaining the network and its portfolio of services.

## Introduction

Without oversight, private health care markets will reliably disappoint social expectations for service quality and their accessibility to the poor [1]. To satisfy concerns for quality and accessibility, some entity will need to intervene as a steward of private health care markets and their stewardship will incur costs. Spending money to improve service quality and offer access to the poor will often fail to register impact in terms of conventional cost-effectiveness metrics such as dollars per disability or quality adjusted life year. Some would argue that health systems improvements that cannot be shown to improve population health should not receive priority. In contrast, the United Nations states that high quality health facilities are an essential element of the right to health [2]. According to the UN, governments should improve health care quality to guarantee citizen rights, not just because quality improvement is efficient nor to the extent that quality saves lives. Much of economic evaluation remains consequentialist and excludes from analysis any preferences patients might have about the processes that the health system uses to achieve health outcomes [3]. Beyond welfarism, and extra-welfarism is the reality that the quality of services matters to patients and equality of access matters and patients and policy makers are willing to spend resources to improve these aspects of the health system.

In areas where government stewardship is lacking, non-governmental organizations (NGOs) find themselves assuming a role in improving health service quality. Typical medical NGOs in low income countries hire most of their medical care providers and keep them on a payroll and directly supervise service quality. There are other NGOs, for example social franchises, that gather private sector providers in a network and improve service quality without directly hiring providers [4,5].

As more donors face situations where health system stewardship requires creative solutions they want to be sure their investments serve the purposes intended. Little is known about what it costs to improve quality and improve service access in the private sector of low income countries. Existing studies in Ethiopia and Pakistan show that social franchises can provide higher quality than non-franchised private clinics, but with higher costs per client treated for family planning services [6].

By following the flow of funds in a social franchise that is explicitly focused on service quality and reaching the poor, we can assess what it will cost to address some of the most dire deficiencies in the health system. Improving service quality in any system will require extra resources—until these resources are defined and financed, progress will be difficult.

The objective of this paper is to measure the cost of health care quality improvements by focusing on the operations of the Sun Quality and Sun Primary health networks operated by Population Services International (PSI) in Myanmar. For every dollar spent by patients acquiring medical care in a social franchise, our analysis will tally the total dollars spent by the franchise on subsidizing that service and regulating its quality. Ordinarily, customers pay more than the wholesale price. Because social franchises are not like ordinary retail markets, our analysis will identify the areas where patients pay less than wholesale.

## Background

The sale of medical care episodes in competitive private markets allows one to observe prices paid by buyers. Competitive forces and the absence of insurance programs in Myanmar make it plausible that the equilibrium prices for private medical care observed in Myanmar reflect what patients are willing to pay to purchase medical care episodes and what suppliers can comfortably afford to sell them for. This market solution to medical care provision succeeds in balancing supply with demand. It is well known to fail to provide quality and to protect the poor [7].

Protecting the poor requires subsidies and improving quality requires resources. To improve and monitor the quality of professional services there must be some type of data collection exercise. Somehow or other information about services provided needs to be collected, inspected, and feedback must be given to the provider with advice and/or incentives for improvement. Low capacity, competing priorities, and political opposition hinders efforts to achieve adequate quality regulation of the private practice of medical care. The problem is global [8]. The social franchising solution used by PSI/Myanmar is to use the commodity distribution network to also serve as way to monitor and improve the quality of service.

In a social franchise, a network of private providers is created. There are two separate networks in PSI Myanmar: Sun Quality Health (SQH) network is primarily physicians and advanced health professionals while Sun Primary Health (SPH) providers typically have a lower level of training and often operate as mobile providers in an effort to reach those most at risk and in rural areas. In the franchised delivery model “Sun Field Leaders” serve as the quality supervisors and the distributors of subsidized commodities. The subsidies sustain the continued voluntary membership and they encourage providers to devote time and space to services they might not otherwise provide. Sometimes the providers devote 100% of their activities to services associated with the franchise, sometimes, as in a fractional franchise, the providers offer other services or products besides those of the franchise. PSI/Myanmar is an example of a fractional franchise. Private providers in the franchise also agree to receive regular visits by the franchisor dedicated to monitoring and improving service quality in the use of these commodities [4,5]. The structural design of a social franchise allows one to determine separately the costs of solving public policy problems of subsidizing access and enhancing quality. We compare these costs to the private costs that households expend to solve their private problem of acquiring medical services. This analysis can thus produce estimates of the ratio of costs expended to supply the publicly valued aspects of medical care episodes to the costs that patients routinely pay to purchase privately valued episodes.

In 2009, PSI’s franchising department supported a team of over 1,700 private providers currently enrolled in PSI’s Network. There were 1006 and 741 in the Sun Quality and Sun Primary Health Networks, respectively in 2009. Social franchising activities were supported in part by four other departments within PSI’s organizational structure. (1) Social Marketing (423 staff); (2) Strategic Information (36 staff); (3) Finance (136 staff); and (4) Program Support (125 staff). These departments were supported by international technical advisors, senior advisors, an executive office team, the finance monitoring unit, and a special advocacy team, all of which fall under the leadership of PSI-Myanmar’s country director. Most critically, the social marketing department works to provide support to product branding as well communications, whilst strategic information facilitates the collection of routine data on products sales, distribution, and incentives.

There is emerging evidence that the PSI network of providers in Myanmar is able to improve service quality. The SPH members can attend two initial 3-day trainings, one month apart and then receive regular visits by field leaders and stocks of rapid diagnostic test kits and artemisin containing treatment packs. In an evaluation of PSI/Myanmar’s effects on quality of care, a team of researchers validated [9] and applied the use of an observed simulated patient to assess the management of a child with malaria by providers before and after adopting the PSI-malaria line of service [10]. They found that workers enrolled in PSI’s malaria program achieved and sustained better performance in managing malaria for over 12 months [10]. There is also evidence that Myanmar’s PSI network is able to target the poor. In urban areas, TB patients at SQH clinics were more likely to be in the poorest quintile compared to the general TB positive urban population [11]. These promising results motivate the present investigation into the cost of PSI/Myanmar’s service improvement and delivery model.

## Methods

### Data

Since its inception in 2001, Population Services International (PSI) Myanmar’s social franchising program has expanded from a focus on reproductive health to include new providers and disease treatment lines for malaria and STIs (2003), tuberculosis (2004), and diarrhea and pneumonia in children under 5 (2008) [12]. The tuberculosis program in PSI allows TB patients to obtain their directly observed treatment short course (DOTS) under the supervision of the private provider. The economics of the TB program are very different from the other lines of service: patients pay no fees to the provider, providers receive capitated payments from the TB program, drugs are procured by the national TB program. These special features make the TB program sufficiently different that it will not be considered here.

**Figure 1:**
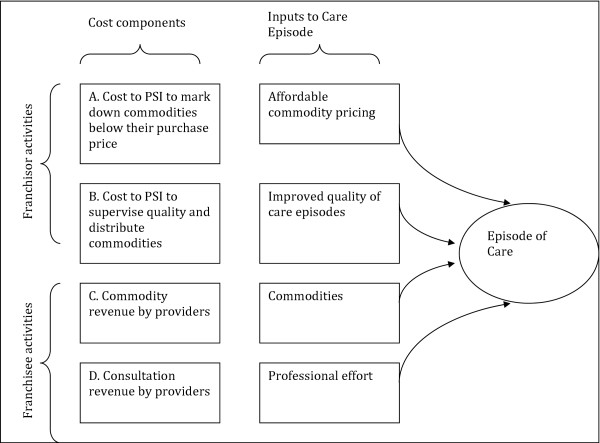
**Conceptual framework.** The top two rows relate to costs and inputs supplied by PSI as franchisor. The bottom two rows are costs and inputs supplied by private health care providers. The “inputs” supplied by PSI are price reductions as well as oversight of the distribution network and final medical care episode. This oversight occurs under the agency of franchise officers who visit each private provider on a monthly basis. The inputs supplied by private providers are medical commodities and professional effort.

Costs were estimated using an ingredients approach for 2007 to 2009 through a retrospective review of financial reports maintained by PSI-Myanmar’s Finance Department in Yangon. All figures were presented in 2009 USD, corresponding to the final year of analysis. The PSI finance department in Yangon, Myanmar provided access to official Government of Myanmar monthly exchange rates for the 2007–2009 analytic time horizon. Period averages were generated for each year and utilized to Kyats to US dollars. Costs incurred during years prior to 2009 were converted to 2009 dollars using local consumer price indices obtained from the IMF's International Financial Statistics Yearbook. The financial accounting system prior to 2007 was maintained on a different system of ledgers making it difficult to reconcile expenditure categories. Emphasis was placed upon the identification of major activities associated with the scale-up and sustained implementation of SQH and SPH delivery channels and new lines of services. There are four major elements to the cost model. Figure 1 breaks them into two elements at the level of Franchisor (PSI) and two elements at the level of the Franchisee (Private provider). These are as follows: A. Costs to PSI headquarters to acquire medical commodities; B. Costs to PSI associated with the packaging, distribution, and service monitoring related to products channeled through the franchise network C. Revenue received by providers in return for product sales; D. Revenue received by SPH/SQH providers in the delivery of professional services.

#### Procurement of commodities and supplies

PSI/Myanmar’s procurement department communicates with international and local suppliers. They also are attuned to the drug registration processes at the Myanmar Food and Drug Administration (FDA). PSI proactively obtains clearance from the Myanmar government to import commodities. PSI-Myanmar’s procurement department is comprised of a staff of 9 including access to an international advisor, program support director, senior procurement manager and five additional support staff located within three sub-units: (1) local procurement; (2) quality control; and (3) international procurement. In addition to country-level procurement in Myanmar, PSI’s global headquarters in Washington, DC maintains a separate procurement department that supports country-level requests in excess of US$10,000. For these larger orders, PSI-Washington coordinates internationally competitive bidding, product quality control testing and shipment to Myanmar. The Washington office obtains globally competitive prices and takes advantage of bulk-purchasing power, often sourcing commodities in Asia. The overhead for global procurement from Washington is a cost shared by dozens of countries and millions of people and would not be altered by the presence or absence of a Myanmar operation, so it is omitted from analysis.

Once products are procured, PSI sells medical commodities (tuberculosis treatment being the sole exception) to network providers based on pre-negotiated prices. Franchise members’ commodity prices are highly subsidized in order to interest providers in offering services that they would not otherwise offer and to sustain their voluntary participation in the network.

#### Costs associated with the packaging and distribution of commodities

Once procured, commodities are packaged in Yangon-based warehouses. The packaging utilized is designed by PSI’s marketing and communications department and produced locally in Myanmar. Financial records were reviewed to determine costs associated with the packaging, re-sale and distribution of products in Myanmar. In the design of social franchises product distribution to clinics is inseparable from the supervision of service quality during monthly visits to each member. PSI/Myanmar’s franchise officers are all physicians and they spend several hours during each visit reviewing clinical records, identifying complex cases, and discussing case management with providers.

Unit cost analysis was done and the results presented. An ingredients based model of the franchise services and those of supporting PSI-departments was utilized to allocate overhead costs. As part of this activity, key informant interviews with staff in all departments were carried out to identify the full portfolio of products procured, supporting materials generated, and appropriate proportion attributable to the franchising component of PSI’s overall portfolio. Interviews were carried out with the following staff of PSI Myanmar Office 1) Director; 2)Chief financial officer; 3) Chief procurement officer; 4)Current chief of franchising operations; 5) Past chief of franchising; 6) Chief of research; 7) Current chief of social marketing. The interviews were used to determine the appropriate lines of service to study and how to allocate administrative overhead to the various lines of service.

The documents reviewed consisted of electronic ledgers of all payments for 2007 to 2009 and log books of all commodities distributed to franchise members for the same period. The ledgers and log-books contained the financial data that form the substance of the unit cost estimates we present. The PSI/Myanmar office had conducted a time –motion study in an attempt to measure the contribution of franchise officers’ time across the various product lines and across administrative work vs. quality supervision.

Allocating overhead from the administrative units at headquarters was based on the proportion of commodity costs attributed to the franchising program. Indirect costs associated with building and administrative costs were allocated at approximately 33% to the franchising program. This percentage reflects the total proportion of PSI’s annual franchising budget out of the total annual operating budget for all programs. Direct costs associated with transportation, staff salaries, program incentives, etc. were obtained through an intensive review of PSI’s financial records and cross-checked through in-depth interviews with PSI staff to ensure the exhaustive inclusion of all potential expenditures.

#### Revenue received by PSI headquarters in return for commodities

We estimated the total revenue PSI/Myanmar received in return for the sale of commodities to its members. Data on total products sold and funds received were obtained from PSI’s management records.

#### Costs incurred by SPH/SQH providers in the delivery of commodities and services

Providers are allowed by the franchise to sell most of the purchased products that they have acquired at subsidized prices. There are exceptions: for example, there can be no charges for TB drugs. Furthermore it is recommended that providers adhere to PSI’s pricing list. Data on the revenue obtained by providers through the sale of their products are not collected, however we do know how many commodities were sold and their suggested retail price. Providers agree to a PSI price and fee schedule in billing for the commodities and professional services that are required to accompany the PSI commodities, and they post the fees on the wall of the facility. With data on the number of visits we use the fee schedule to benchmark the amount of retail revenue private providers would obtain from selling commodities distributed by PSI. We compare administrative costs and subsidies to this benchmark.

## Results

Table 1 demonstrates the total cost for all PSI procurement activities across all programs for 2007–2009. As shown in Table 1 the procurement costs for commodities in the franchising program cost a total of $2.15 million in 2009. Table 1 only shows the costs of com-modities distributed through private providers in SQH and SPH networks. PSI Myanmar is able to recover $125,541 from the SQH and SPH members who pay marked down prices for commodities. Thus its total injection of financing into supporting lower prices of commodities totals $2.02 million in 2009.

**Table 1:** Procurement costs for all major PSI programs by major disease areas for 2009 in USD

	**2009**	**2008**	**2007**
RH	$905,577	$921,684	$807,870
Malaria	$1,067,708	$410,445	$347,118
STI	$22,731	$42,868	$39,485
TB Suspect	$-	$-	$-
Pneumonia	$26,463	$15,166	$-
Diarrhea*	$126,356	$611	$696
TOTAL	$2,148,835	$1,390,775	$1,195,169

*Prior to 2009 diarrhea program was a water treatment product called Waterguard, this was joined by ORS products in 2009 as shown in Additional file 1: Table S4.

Table 2 tracks the commodity subsidies provided by PSI/ Myanmar. Column A shows the total that PSI Myanmar spends to procure commodities and subsequently column B shows the payments received by its members for these commodities. For 2009, $2.15 million in commodities were devoted to these 5 categories of service in the franchise program. Overall franchise members are receiving commodities at a 94% discount from prices on the world market. The discounts are somewhat less for the antibiotics used for STIs and pneumonia. Based on recommended sales prices, we estimate that the providers generated revenue of $648,000 from the medical visits associated with these commodities. The pricing structure thus passed along $1.38 million in subsidized medical commodities to the patients of Myanmar, with most of the subsidy being generated by commodities for malaria. As shown in Table 2, patients do not receive subsidized pricing for STI and pneumonia commodities. For STIs and pneumonia, the Sun network providers are behaving like ordinary retailers and marking up wholesale prices. The antibiotic pricing policy was negotiated as an incentive to persuade franchise members to integrate STI treatment into their practice. The higher markups are also enabled both by a robust demand for antibiotics in Myanmar and by competitive advantages that PSI’s procurement department has achieved in securing lower antibiotic prices than those available to other private sector health providers.

**Table 2:** Tracking the flow of subsidies for commodities in PSI Myanmar

**Flows of funds in PSI/Myanmar(Average for 2007-2009)**							
	**Procure-ment cost for PSI Myanmar head-quarters**	**Revenue from members to headquarters**	**Subsidy provided by PSI**	**%subsidy provided by PSI**	**Revenue to retailer**	**Subsidy for commodity given to patient**	**%subsidy received by patient**
	[A]	[B]	[A-B]	[A-B]/A	C = (Sale Price × Volume)	[D = A-(B + C)]	[D/A]
RH	$878,377	$156,463	$721,914	82%	$347,589	$374,325	42%
Malaria	$608,424	$27,689	$580,734	93%	$79,042	$501693	77%
STI	$35,028	$10,830	$24,198	64%	$60,151	$(35,954)	-123%
Pneumonia	$20,815	$11,462	$9,354	35%	$64,304	$(54,951)	-237%
Diarrhea	$42,554	$3,745	$38,809	53%	$4,229	$34,580	34%
**Total 2007-2009**	**$4,734,779**	**$619,106**	**$4,115,672**	**87%**	**$1,601,641**	**$2,514,030**	**53%**

Table 3 shows the total costs of maintaining the franchise system to check provider quality and distribute commodities at $2.09 million in 2009. Personnel costs account for 32% of the total with allocated vehicles and transport accounting for 21% of the total for 2009. The share of PSI’s overhead allocated to its franchising division is $180,000 and accounts for 9% of the cost. Note that services are growing from roughly $750,000 in 2007 to $2 million in 2009. (Additional file 1: Table S4 shows a growth in the number of SPH providers from 137 in 2008 to 858 in 2010. Additional file 2: Table S5 shows a growth in the number of providers offering reproductive health services in the SQH network from 710 in 2007 to 930 in 2009). One implication is that some of the spending is being incurred for start up costs of new product lines and new service areas. PSI/Myanmar has shown annual growth since its inception. It is too early to say whether the growth and expansion costs are exceptional or part of the nature of the organization.

**Table 3:** **Costs of packaging**, **distribution**, **and quality supervision at PSI Myanmar headquarters**

	**2009**	**%**	**2008**	**%**	**2007**	**%**
Personnel	$666,045	32%	$379,465	30%	$294,390	19%
Refresher training and events	$234,530	11%	$80,224	6%	$56,952	4%
Vehicle/Transport	$429,980	21%	$249,532	20%	$121,615	13%
Provider reimbursement and incentives	$520,701	25%	$251,300	20%	$213,350	13%
Information education communication	$61,436	3%	$44,319	3%	$7,116	37%
Building Costs	$180,502	9%	$267,680	21%	$64,665	14%
**TOTAL**	**$2,093,195**		**$1,272,519**		**$758,089**	

**Figure 2:**
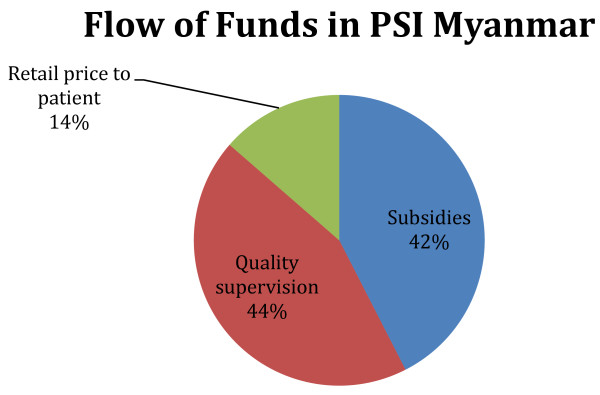
**Allocation of funds in a social franchise.** For every dollar spent on a medical care episode, the patient is spending 14 cents at retail, the social franchise is spending 42 cents to achieve price reductions below the acquisition cost, and spending 44 cents to supervise the quality of care.

In the franchised health care system operated by PSI Myanmar, PSI’s outlay of resources to achieve lower commodity prices is $2.02 million (=$2,148,835 spent minus $125, 823 in revenue from providers) and the outlay to supervise quality and to package and distribute the commodities is $2.09 million (Table 3 column 1) for a total of nearly $4.1 million spent on its social franchise operations in 2009. The $4.1 million was spent to add value to an estimated 1.5 million medical care episodes occurring in the private sector with a total cost to patients of $647,000. Taking the ratio of $4.1 million to the number of episodes (1.5 million) one derives that PSI’s social franchise spending amounts to $2.7 per medical care episode. Taking the ratio of $4.1 million to patient out of pocket spending on these 5 commodities ($648,000) and one derives a ratio of $6.32 spent by PSI per patient dollar spent. Figure 2 shows this data in terms of proportions. For every dollar spent on a medical care episode, the patient is spending 14 cents at retail, the social franchise is spending 42 cents to achieve price reductions below the acquisition cost, and spending 44 cents to supervise the quality of care.

The commodities needed for these 1.5 million consultations would have cost $2.15 million at globally competitive prices. In the absence of PSI’s social franchise subsidies, most prices would have been higher for the patients served in these private facilities without subsidies, it is reasonable to assume that fewer providers would have offered services and fewer patients would have accessed services. Furthermore in the absence of PSI’s social franchise, the quality of unsupervised consultations would probably have been lower. Studies are emerging that measure the degree of quality improvement and the degree to which the subsidies help the poor in PSI Myanmar [10,11].

## Discussion

Our analysis shows that in order for PSI to achieve subsidized prices and quality supervision in its networks of private providers it must spend money on both subsidies and oversight. For every dollar spent on commodities by the people of Myanmar at PSI’s SQH clinics and SPH providers in 2009, donors (via PSI) spent $6.32 to achieve important goals in public health—quality assurance and access to services that the poor may not have otherwise been able to achieve.

Policymakers cite many reasons to be unhappy with the unregulated provision of private health care. Until our analysis, little has been known about the costs and flow of funds in efforts to improve private sector health services. We estimate that PSI Myanmar is spending $2.7 per private sector medical care episode in efforts to increase quality, affordability, accessibility, and usage in its network. We are the first to benchmark this expenditure for Myanmar, and so we cannot yet say if this spending should be more or less. However, in Myanmar patients spending $1 for a medical care episode at a PSI franchised facility are having their dollar matched by $6.32 in spending by PSI and its sponsors in an effort to achieve policy goals. Private providers that don’t subsidize their medical services are viable, but the types of services studied in this paper were provided with $6.32 extra per $1.00 of commodities in order to achieve better quality and subsidies for the poor. Going forward, policy makers will need to determine how to finance investments in quality and lower prices for the poor and how to achieve these goals efficiently.

## Competing interest

The authors declared that they have no competing interest.

## Authors’ contribution

DB and DM designed the study. DB, AL, MB, JH, and MZ carried out data collection. DB and AL conducted data analysis. All authors participated in interpreting the findings and drafting the paper. All authors read and approved the final manuscript.

## Author’s information

Social Franchising Research Team (in alphabetical order): Ohnmar Htun, Jayne Rowan, Tin Aung, Wadi Hlaing Tun, Yin Yin Thein, Suesandau Htun.

Marc Theuss, Matt Boxshall, John D. Hertherington and Dominic Montaga are staff member of Population Services International. The authors are responsible for the views expressed in this publication and they do not necessarily represent the decisions, policy or views of Population Services International.

## Supplementary Material

Additional file 1: Table S4 Summary of SQH and SPH providers and product lines from 2001-2010. Click here for file

Additional file 2: Table S5 Estimated annual consultations by provider. Click here for file
