# Supplementary material for: The cost of service quality improvements: tracking the flow of funds in social franchise networks in Myanmar
Source: Cost Eff Resour Alloc. 2013 Jul 4;11:14. doi: 10.1186/1478-7547-11-14 (PMC3717111; doi:10.1186/1478-7547-11-14)
Supplement: Supplementary file 1 [file 1478-7547-11-14-S1.doc]

Appendix Table 4. Summary of SQH and SPH providers and product lines from 2001-2010

	2001	2002	2003	2004	2005	2006	2007	2008	2009	2010
Health area expansion	Reproductive Health		Malaria STIs	TB		Water guard	Pneumonia	Reproductive Health (LTM)	Orasal kit	PICT
Number of new products added	3	5	14	17	17	18	23	24	26	28
New product names	Oral and 3-month injectable contraceptives; male condom	1-month injectable contraceptive; emergency contraceptive	STI treatment; IUD; Malaria diagnosis and treatment	3 DOTS kits		Waterguard	Pneumonia treament (4 trimox); Female condom	Pregnancy test	Jardelle; Zinc/ ORS	HIV tests--Determine and Unigold
No of SQH (overall)	73	124	331	467	559	707	797	879	1006	1169
Only one health area	73	124	166	222	148	207	154	140	157	197
Two health areas			175	189	246	253	200	183	155	204
Three health areas			9	88	179	230	218	199	236	262
Four health areas				8	35	97	207	229	267	291
Five health areas							131	266	358	383
No of new townships	23	21	47	36	32	55	49	48	70	62
Male:Female (yearly)	2.8:1	1.3:1	1.6:1	3.4:1	3.8:1	2.4:1	2.6:1	2.1:1	1.4:1	1.9:1
No of SPH (overall)								137	741	858
Three health areas								63	12	8
Four health areas								73	433	469
Five health areas									370	485
No of new townships								6	27	15
Male:Female (yearly)								0.33:1	0.47:1	0.51:1
