# Supplementary material for: The cost of service quality improvements: tracking the flow of funds in social franchise networks in Myanmar
Source: Cost Eff Resour Alloc. 2013 Jul 4;11:14. doi: 10.1186/1478-7547-11-14 (PMC3717111; doi:10.1186/1478-7547-11-14)
Supplement: Supplementary file 2 [file 1478-7547-11-14-S2.doc]

Appendix Table 5. Estimated annual consultations by provider

		RH		Malaria		STI		TB Suspect		Pneumonia		Diarrhea	
		SQH	SPH	SQH	SPH	SQH	SPH	SQH	SPH	SQH	SPH	SQH	SPH
	2009												
Providers	Total no. providing service	930	785	550	424	742		533	393	666	595		782
	% of total subscribers	79%	96%	47%	52%	63%		45%	48%	57%	73%		95%
Consultations	Total annual all providers	1,039,378	58,394	104,290	20,573	42,780		15,352	2,025	173,814	9,437		39,301
	Mean per provider per year	1,118	74	190	49	80		29	5	261	16		50
Estimated revenue per patient	Mean	$ 0.36	$ 0.19	$ 0.74	$ 0.29	$ 1.46		$ -	$ -	$ 0.61	$ 0.39		$ 0.32
	High	$ 0.49	$ 0.19	$ 0.97	$ 0.97	$ 1.46		$ -	$ -	$ 0.78	$ 0.78		$ 0.34
	Low	$ 0.19	$ 0.19	$ 0.29	$ 0.19	$ 1.46		$ -	$ -	$ 0.49	$ 0.19		$ 0.34
Estimated annual revenue per	Mean	$ 398	$ 14	$ 140	$ 14	$ 116		$ -	$ -	$ 158	$ 6		$ 16
	High	$ 542	$ 14	$ 185	$ 47	$ 116		$ -	$ -	$ 203	$ 12		$ 17
	Low	$ 217	$ 14	$ 55	$ 9	$ 116		$ -	$ -	$ 127	$ 3		$ 17
Total revenue all providers	**Mean**	**$ 369,980**	**$ 11,338**	**$ 76,778**	**$ 5,992**	**$ 62,297**		**$ -**	**$ -**	**$ 105,463**	**$ 3,665**		**$ 12,400**
	High	$ 504,519	$ 11,338	$ 101,246	$ 19,972	$ 62,297		$ -	$ -	$ 134,992	$ 7,329		$ 13,354
	Low	$ 201,808	$ 11,338	$ 30,374	$ 3,994	$ 62,297		$ -	$ -	$ 84,370	$ 1,832		$ 13,354
	2008												
Providers	Total no. providing service	783	112	480	64	536		430	22	392	95		38
	% of total subscribers	79%	80%	48%	46%	54%		43%	16%	40%	68%		27%
Consultations	Total annual all providers	958,967	5,750	108,408	834	42,780		10,331	45	31,732	586		914
	Mean per provider per year	1,225	51	226	13	80		24	2	81	6		24
Estimated revenue per patient	Mean	$ 0.36	$ 0.19	$ 0.74	$ 0.29	$ 1.46		$ -	$ -	$ 0.61	$ 0.39		$ 0.32
	High	$ 0.49	$ 0.19	$ 0.97	$ 0.97	$ 1.46		$ -	$ -	$ 0.78	$ 0.78		$ 0.34
	Low	$ 0.19	$ 0.19	$ 0.29	$ 0.19	$ 1.46		$ -	$ -	$ 0.49	$ 0.19		$ 0.34
Estimated annual revenue per	Mean	$ 436	$ 10	$ 166	$ 4	$ 116		$ -	$ -	$ 49	$ 2		$ 8
	High	$ 594	$ 10	$ 219	$ 13	$ 116		$ -	$ -	$ 63	$ 5		$ 8
	Low	$ 238	$ 10	$ 66	$ 3	$ 116		$ -	$ -	$ 39	$ 1		$ 8
Total revenue all providers	**Mean**	**$ 341,357**	**$ 1,116**	**$ 79,810**	**$ 243**	**$ 62,297**		**$ -**	**$ -**	**$ 19,254**	**$ 228**		**$ 288**
	High	$ 465,487	$ 1,116	$ 105,243	$ 810	$ 62,297		$ -	$ -	$ 24,645	$ 455		$ 311
	Low	$ 186,195	$ 1,116	$ 31,573	$ 162	$ 62,297		$ -	$ -	$ 15,403	$ 114		$ 311
	2007												
Providers	Total no. providing service	710		500		462		372					
	% of total subscribers	78%		55%		51%		41%					
Consultations	Total annual all providers	896,088		100,927		38,360		10,143					
	Mean per provider per year	1,262		202		83		27					
Estimated revenue per patient	Mean	$ 0.36		$ 0.74		$ 1.46		$ -					
	High	$ 0.49		$ 0.97		$ 1.46		$ -					
	Low	$ 0.19		$ 0.29		$ 1.46		$ -					
Estimated revenue per provider	Mean	$ 449		$ 149		$ 121		$ -					
	High	$ 613		$ 196		$ 121		$ -					
	Low	$ 245		$ 59		$ 121		$ -					
Total revenue all providers	Mean	**$ 318,974**		**$ 74,302**		**$ 55,860**		**$ -**					
	High	$ 434,965		$ 97,981		$ 55,860		$ -					
	Low	$ 173,986		$ 29,394		$ 55,860		$ -					
